# *catena*-Poly[[diaqua­(4,4′-trimethyl­ene­dipyridine-κ*N*)cobalt(II)]-μ-tereph­thalato-κ^2^ *O* ^1^:*O* ^4^]

**DOI:** 10.1107/S1600536808043584

**Published:** 2009-01-08

**Authors:** Xu-Liang Qi

**Affiliations:** a Liaocheng Vocational and Technical College, LiaoCheng 252000, ShanDong, People’s Republic of China

## Abstract

The title compound, [Co(C_8_H_4_O_4_)(C_13_H_14_N_2_)_2_(H_2_O)_2_]_*n*_, was obtained by the reaction of CoCl_2_, 4,4′-trimethyl­enedipyridine and terephthalic acid in a 1:1:1 ratio. The octa­hedrally coordinated cobalt ions are bridged by 4,4′-trimethyl­enedipyridine ligands, generating a chain. These chains are further linked by O—H⋯O and O—H⋯N hydrogen bonds, giving a three-dimensional network.

## Related literature

For a related structure, see Manna *et al.* (2005 ▶).
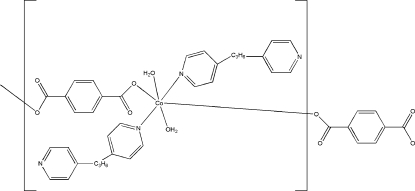


## Experimental

### 

#### Crystal data

[Co(C_8_H_4_O_4_)(C_13_H_14_N_2_)_2_(H_2_O)_2_]
*M* *_r_* = 655.60
Monoclinic, 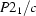

*a* = 11.232 (2) Å
*b* = 9.3784 (19) Å
*c* = 15.182 (3) Å
β = 96.19 (3)°
*V* = 1589.9 (5) Å^3^
*Z* = 2
Mo *K*α radiation
μ = 0.59 mm^−1^
*T* = 293 (2) K
0.20 × 0.14 × 0.08 mm

#### Data collection

Bruker SMART 1K CCD area-detector diffractometer
Absorption correction: multi-scan (*SADABS*; Sheldrick, 2004 ▶) *T*_min_ = 0.891, *T*_max_ = 0.954
8438 measured reflections
2726 independent reflections
2167 reflections with *I* > 2σ(*I*)
*R*_int_ = 0.068

#### Refinement

*R*[*F* ^2^ > 2σ(*F* ^2^)] = 0.070
*wR*(*F* ^2^) = 0.179
*S* = 1.13
2726 reflections
207 parameters
H-atom parameters constrained
Δρ_max_ = 1.05 e Å^−3^
Δρ_min_ = −0.62 e Å^−3^

### 

Data collection: *SMART* (Bruker, 2001 ▶); cell refinement: *SAINT* (Bruker, 2001 ▶); data reduction: *SAINT*; program(s) used to solve structure: *SHELXS97* (Sheldrick, 2008 ▶); program(s) used to refine structure: *SHELXL97* (Sheldrick, 2008 ▶); molecular graphics: *SHELXTL* (Sheldrick, 2008 ▶); software used to prepare material for publication: *SHELXTL* and local programs.

## Supplementary Material

Crystal structure: contains datablocks I, global. DOI: 10.1107/S1600536808043584/bt2833sup1.cif

Structure factors: contains datablocks I. DOI: 10.1107/S1600536808043584/bt2833Isup2.hkl

Additional supplementary materials:  crystallographic information; 3D view; checkCIF report

## Figures and Tables

**Table 1:** Hydrogen-bond geometry (Å, °)

*D*—H⋯*A*	*D*—H	H⋯*A*	*D*⋯*A*	*D*—H⋯*A*
O3—H3*A*⋯N2^i^	0.93	1.90	2.820 (5)	171
O3—H3*B*⋯O2^ii^	1.03	1.71	2.704 (4)	161

Symmetry codes: (i) 
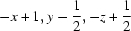
; (ii) 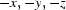.

**Fig. 1.**
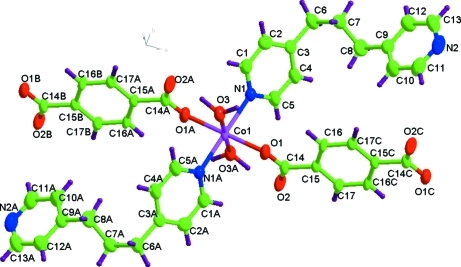
An ORTEP view of the Co centre and its surrounding ligands with 50% displacement ellipsoids for non-H atoms. Symmetry code: (A) -x,-y,-z

**Fig. 2.**
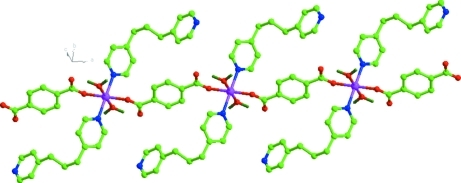
Partial packing diagram of the title compound.

## supplementary crystallographic information

### Comment

Co  is six-coordinated by two terephthalate O atoms, two N atoms of 4,4'-trimethylenedipyridines and two  water molecules in a distorted octahedral fashion. The Co—O and Co—N bond lengthes are in the normal range. The 4,4'-trimethylenedipyridine and terephthalate ligands adopt bidentate coordinated modes. As shown in Fig. 2, a   chain structure is formed. These chains  are further linked by   O—H···O and O—H···N hydrogen bonds  to generate a three-dimensional network.

### Experimental

CoCl_2_(1.0 mmol), terephthalic acid  (1 mmol), and 4,4'-trimethylenedipyridine (1 mmol) were dissolved in water (10 ml). The solution was heated in a 25 ml Teflon lined reaction vessel at 433 K for *ca* 3 days and then cooled to room temperature. Purple crystals were obtained in a yield of 65%.

### Refinement

All non-water H atoms were positioned geometrically and refined by using a riding model with C—H = 0.93–0.96 Å and with *U*_iso_(H) = 1.2 times *U*_eq_(C), the water H atoms are firstly found, then refined freely.

### Crystal data

**Table:**

[Co(C_8_H_4_O_4_)(C_13_H_14_N_2_)_2_(H_2_O)_2_]	*F*(000) = 686
*M**_r_* = 655.60	*D*_x_ = 1.369 Mg m^−^^3^
Monoclinic, *P*2_1_/*c*	Mo *K*α radiation, λ = 0.71073 Å
*a* = 11.232 (2) Å	Cell parameters from 3657 reflections
*b* = 9.3784 (19) Å	θ = 1.4–27.9°
*c* = 15.182 (3) Å	µ = 0.59 mm^−^^1^
β = 96.19 (3)°	*T* = 293 K
*V* = 1589.9 (5) Å^3^	Block, purple
*Z* = 2	0.20 × 0.14 × 0.08 mm

### Data collection

**Table:**

Bruker SMART 1K CCD area-detector diffractometer	2726 independent reflections
Radiation source: sealed tube	2167 reflections with *I* > 2σ(*I*)
graphite	*R*_int_ = 0.068
Detector resolution: 8.192 pixels mm^-1^	θ_max_ = 25.0°, θ_min_ = 1.8°
Thin–slice ω scans	*h* = −11→13
Absorption correction: multi-scan (*SADABS*; Sheldrick, 2004)	*k* = −8→11
*T*_min_ = 0.891, *T*_max_ = 0.954	*l* = −17→18
8438 measured reflections	

### Refinement

**Table:**

Refinement on *F*^2^	Primary atom site location: structure-invariant direct methods
Least-squares matrix: full	Secondary atom site location: difference Fourier map
*R*[*F*^2^ > 2σ(*F*^2^)] = 0.070	Hydrogen site location: inferred from neighbouring sites
*wR*(*F*^2^) = 0.179	H-atom parameters constrained
*S* = 1.13	*w* = 1/[σ^2^(*F*_o_^2^) + (0.0794*P*)^2^ + 1.059*P*] where *P* = (*F*_o_^2^ + 2*F*_c_^2^)/3
2726 reflections	(Δ/σ)_max_ < 0.001
207 parameters	Δρ_max_ = 1.05 e Å^−^^3^
0 restraints	Δρ_min_ = −0.62 e Å^−^^3^

### Fractional atomic coordinates and isotropic or equivalent isotropic displacement parameters (Å^2^)

**Table:**

	*x*	*y*	*z*	*U*_iso_*/*U*_eq_	
Co1	0.0000	0.0000	0.0000	0.0323 (3)	
O1	0.1776 (3)	−0.0428 (3)	−0.0026 (2)	0.0398 (8)	
O2	0.2160 (3)	−0.0377 (4)	−0.1445 (2)	0.0560 (10)	
O3	0.0130 (3)	−0.0481 (3)	0.1394 (2)	0.0409 (8)	
N1	0.0522 (3)	0.2209 (4)	0.0279 (2)	0.0369 (9)	
N2	0.7841 (4)	0.5278 (5)	0.2442 (3)	0.0562 (12)	
C1	0.0265 (4)	0.2912 (5)	0.1005 (3)	0.0451 (11)	
H1	−0.0263	0.2493	0.1359	0.054*	
C2	0.0747 (4)	0.4234 (5)	0.1253 (3)	0.0479 (12)	
H2	0.0544	0.4674	0.1765	0.057*	
C3	0.1530 (4)	0.4901 (5)	0.0739 (3)	0.0378 (10)	
C4	0.1764 (4)	0.4190 (5)	−0.0026 (3)	0.0443 (11)	
H4	0.2263	0.4604	−0.0405	0.053*	
C5	0.1259 (4)	0.2869 (5)	−0.0226 (3)	0.0449 (12)	
H5	0.1441	0.2415	−0.0739	0.054*	
C6	0.2111 (4)	0.6317 (5)	0.1003 (4)	0.0541 (14)	
H6A	0.1600	0.6837	0.1367	0.065*	
H6B	0.2183	0.6877	0.0474	0.065*	
C7	0.3359 (4)	0.6137 (5)	0.1519 (4)	0.0544 (14)	
H7A	0.3618	0.7056	0.1764	0.065*	
H7B	0.3290	0.5496	0.2013	0.065*	
C8	0.4310 (5)	0.5574 (6)	0.0989 (4)	0.0612 (15)	
H8A	0.4361	0.6191	0.0482	0.073*	
H8B	0.4073	0.4634	0.0767	0.073*	
C9	0.5539 (4)	0.5470 (5)	0.1508 (3)	0.0436 (11)	
C10	0.6121 (4)	0.4186 (5)	0.1644 (4)	0.0516 (13)	
H10	0.5755	0.3347	0.1429	0.062*	
H3B	−0.0679	−0.0089	0.1545	0.09 (2)*	
H3A	0.0837	−0.0307	0.1753	0.10 (2)*	
C11	0.7261 (4)	0.4149 (6)	0.2103 (4)	0.0553 (14)	
H11	0.7639	0.3268	0.2176	0.066*	
C12	0.6140 (5)	0.6664 (6)	0.1852 (4)	0.0579 (14)	
H12	0.5787	0.7559	0.1780	0.070*	
C13	0.7273 (5)	0.6521 (6)	0.2307 (4)	0.0602 (15)	
H13	0.7660	0.7341	0.2531	0.072*	
C14	0.2472 (4)	−0.0357 (4)	−0.0632 (3)	0.0364 (10)	
C15	0.3786 (3)	−0.0193 (4)	−0.0300 (3)	0.0314 (9)	
C16	0.4142 (4)	0.0107 (4)	0.0580 (3)	0.0365 (10)	
H16	0.3566	0.0188	0.0974	0.044*	
C17	0.4659 (4)	−0.0292 (4)	−0.0888 (3)	0.0366 (10)	
H17	0.4436	−0.0482	−0.1484	0.044*	

### Atomic displacement parameters (Å^2^)

**Table:**

	*U*^11^	*U*^22^	*U*^33^	*U*^12^	*U*^13^	*U*^23^
Co1	0.0153 (5)	0.0487 (5)	0.0327 (5)	−0.0044 (3)	0.0010 (3)	0.0013 (3)
O1	0.0221 (16)	0.0495 (18)	0.048 (2)	−0.0006 (12)	0.0036 (14)	0.0055 (13)
O2	0.0219 (18)	0.099 (3)	0.046 (2)	−0.0012 (16)	0.0005 (15)	−0.0105 (18)
O3	0.0268 (17)	0.0538 (19)	0.041 (2)	−0.0043 (13)	−0.0021 (14)	0.0020 (14)
N1	0.028 (2)	0.041 (2)	0.042 (2)	−0.0028 (15)	0.0054 (16)	−0.0006 (16)
N2	0.033 (2)	0.089 (4)	0.046 (3)	0.002 (2)	−0.0009 (19)	0.011 (2)
C1	0.035 (3)	0.054 (3)	0.047 (3)	−0.003 (2)	0.009 (2)	−0.001 (2)
C2	0.041 (3)	0.058 (3)	0.044 (3)	0.004 (2)	0.000 (2)	−0.009 (2)
C3	0.024 (2)	0.043 (2)	0.044 (3)	0.0035 (18)	−0.0083 (18)	−0.001 (2)
C4	0.036 (3)	0.048 (3)	0.049 (3)	−0.011 (2)	0.003 (2)	0.003 (2)
C5	0.038 (3)	0.049 (3)	0.049 (3)	−0.009 (2)	0.008 (2)	0.000 (2)
C6	0.034 (3)	0.048 (3)	0.077 (4)	0.000 (2)	−0.009 (2)	−0.011 (2)
C7	0.037 (3)	0.056 (3)	0.068 (4)	0.001 (2)	−0.006 (2)	−0.019 (3)
C8	0.039 (3)	0.078 (4)	0.062 (4)	0.001 (3)	−0.012 (3)	−0.014 (3)
C9	0.032 (3)	0.053 (3)	0.045 (3)	0.003 (2)	−0.002 (2)	−0.002 (2)
C10	0.044 (3)	0.049 (3)	0.062 (4)	−0.006 (2)	0.006 (2)	0.007 (2)
C11	0.041 (3)	0.064 (4)	0.062 (4)	0.012 (3)	0.009 (3)	0.022 (3)
C12	0.046 (3)	0.054 (3)	0.071 (4)	0.004 (2)	−0.009 (3)	−0.005 (3)
C13	0.045 (3)	0.069 (4)	0.064 (4)	−0.016 (3)	−0.008 (3)	−0.006 (3)
C14	0.020 (2)	0.043 (3)	0.046 (3)	−0.0023 (17)	0.0015 (19)	0.0016 (19)
C15	0.018 (2)	0.037 (2)	0.039 (3)	0.0000 (16)	0.0010 (17)	−0.0018 (17)
C16	0.018 (2)	0.054 (3)	0.039 (3)	−0.0029 (18)	0.0095 (18)	−0.0022 (19)
C17	0.023 (2)	0.046 (3)	0.040 (3)	−0.0013 (17)	0.0032 (18)	−0.0042 (18)

### Geometric parameters (Å, °)

**Table:**

Co1—O1^i^	2.039 (3)	C6—H6A	0.9700
Co1—O1	2.039 (3)	C6—H6B	0.9700
Co1—O3^i^	2.154 (3)	C7—C8	1.500 (7)
Co1—O3	2.154 (3)	C7—H7A	0.9700
Co1—N1^i^	2.183 (3)	C7—H7B	0.9700
Co1—N1	2.183 (3)	C8—C9	1.517 (7)
O1—C14	1.271 (5)	C8—H8A	0.9700
O2—C14	1.247 (6)	C8—H8B	0.9700
O3—H3B	1.0283	C9—C10	1.375 (7)
O3—H3A	0.9271	C9—C12	1.381 (7)
N1—C5	1.339 (5)	C10—C11	1.390 (7)
N1—C1	1.342 (6)	C10—H10	0.9300
N2—C11	1.318 (7)	C11—H11	0.9300
N2—C13	1.334 (7)	C12—C13	1.388 (7)
C1—C2	1.389 (7)	C12—H12	0.9300
C1—H1	0.9300	C13—H13	0.9300
C2—C3	1.386 (7)	C14—C15	1.515 (6)
C2—H2	0.9300	C15—C16	1.381 (6)
C3—C4	1.388 (6)	C15—C17	1.398 (6)
C3—C6	1.515 (6)	C16—C17^ii^	1.389 (6)
C4—C5	1.383 (6)	C16—H16	0.9300
C4—H4	0.9300	C17—C16^ii^	1.389 (6)
C5—H5	0.9300	C17—H17	0.9300
C6—C7	1.540 (7)		
			
O1^i^—Co1—O1	180.00 (16)	C7—C6—H6B	109.1
O1^i^—Co1—O3^i^	90.88 (12)	H6A—C6—H6B	107.9
O1—Co1—O3^i^	89.12 (12)	C8—C7—C6	115.3 (5)
O1^i^—Co1—O3	89.12 (12)	C8—C7—H7A	108.5
O1—Co1—O3	90.88 (12)	C6—C7—H7A	108.5
O3^i^—Co1—O3	180.00 (16)	C8—C7—H7B	108.5
O1^i^—Co1—N1^i^	86.99 (12)	C6—C7—H7B	108.5
O1—Co1—N1^i^	93.01 (12)	H7A—C7—H7B	107.5
O3^i^—Co1—N1^i^	91.14 (12)	C7—C8—C9	113.9 (4)
O3—Co1—N1^i^	88.86 (12)	C7—C8—H8A	108.8
O1^i^—Co1—N1	93.01 (12)	C9—C8—H8A	108.8
O1—Co1—N1	86.99 (12)	C7—C8—H8B	108.8
O3^i^—Co1—N1	88.86 (12)	C9—C8—H8B	108.8
O3—Co1—N1	91.14 (12)	H8A—C8—H8B	107.7
N1^i^—Co1—N1	180.0 (2)	C10—C9—C12	116.7 (5)
C14—O1—Co1	133.1 (3)	C10—C9—C8	121.7 (4)
Co1—O3—H3B	100.2	C12—C9—C8	121.6 (4)
Co1—O3—H3A	120.2	C9—C10—C11	119.6 (5)
H3B—O3—H3A	121.8	C9—C10—H10	120.2
C5—N1—C1	116.4 (4)	C11—C10—H10	120.2
C5—N1—Co1	119.8 (3)	N2—C11—C10	124.4 (5)
C1—N1—Co1	123.3 (3)	N2—C11—H11	117.8
C11—N2—C13	115.7 (4)	C10—C11—H11	117.8
N1—C1—C2	123.3 (4)	C9—C12—C13	119.6 (5)
N1—C1—H1	118.4	C9—C12—H12	120.2
C2—C1—H1	118.4	C13—C12—H12	120.2
C3—C2—C1	120.2 (4)	N2—C13—C12	124.0 (5)
C3—C2—H2	119.9	N2—C13—H13	118.0
C1—C2—H2	119.9	C12—C13—H13	118.0
C2—C3—C4	116.3 (4)	O2—C14—O1	125.9 (4)
C2—C3—C6	121.9 (4)	O2—C14—C15	119.4 (4)
C4—C3—C6	121.8 (4)	O1—C14—C15	114.7 (4)
C5—C4—C3	120.2 (4)	C16—C15—C17	118.8 (4)
C5—C4—H4	119.9	C16—C15—C14	120.8 (4)
C3—C4—H4	119.9	C17—C15—C14	120.4 (4)
N1—C5—C4	123.5 (4)	C15—C16—C17^ii^	121.3 (4)
N1—C5—H5	118.2	C15—C16—H16	119.3
C4—C5—H5	118.2	C17^ii^—C16—H16	119.3
C3—C6—C7	112.4 (4)	C16^ii^—C17—C15	119.9 (4)
C3—C6—H6A	109.1	C16^ii^—C17—H17	120.1
C7—C6—H6A	109.1	C15—C17—H17	120.1
C3—C6—H6B	109.1		
			
O1^i^—Co1—O1—C14	−175 (95)	C2—C3—C6—C7	94.5 (5)
O3^i^—Co1—O1—C14	2.6 (4)	C4—C3—C6—C7	−84.7 (6)
O3—Co1—O1—C14	−177.4 (4)	C3—C6—C7—C8	68.8 (6)
N1^i^—Co1—O1—C14	93.7 (4)	C6—C7—C8—C9	177.5 (4)
N1—Co1—O1—C14	−86.3 (4)	C7—C8—C9—C10	118.4 (5)
O1^i^—Co1—N1—C5	−130.3 (3)	C7—C8—C9—C12	−62.9 (7)
O1—Co1—N1—C5	49.7 (3)	C12—C9—C10—C11	−0.5 (7)
O3^i^—Co1—N1—C5	−39.5 (3)	C8—C9—C10—C11	178.3 (5)
O3—Co1—N1—C5	140.5 (3)	C13—N2—C11—C10	−1.2 (8)
N1^i^—Co1—N1—C5	82 (48)	C9—C10—C11—N2	1.1 (8)
O1^i^—Co1—N1—C1	58.2 (4)	C10—C9—C12—C13	0.0 (8)
O1—Co1—N1—C1	−121.8 (4)	C8—C9—C12—C13	−178.7 (5)
O3^i^—Co1—N1—C1	149.1 (4)	C11—N2—C13—C12	0.7 (8)
O3—Co1—N1—C1	−30.9 (4)	C9—C12—C13—N2	−0.2 (9)
N1^i^—Co1—N1—C1	−90 (48)	Co1—O1—C14—O2	−21.8 (7)
C5—N1—C1—C2	−1.9 (7)	Co1—O1—C14—C15	156.4 (3)
Co1—N1—C1—C2	169.9 (3)	O2—C14—C15—C16	167.4 (4)
N1—C1—C2—C3	0.5 (7)	O1—C14—C15—C16	−10.9 (6)
C1—C2—C3—C4	1.5 (7)	O2—C14—C15—C17	−10.2 (6)
C1—C2—C3—C6	−177.7 (4)	O1—C14—C15—C17	171.5 (4)
C2—C3—C4—C5	−2.1 (7)	C17—C15—C16—C17^ii^	−0.8 (7)
C6—C3—C4—C5	177.1 (4)	C14—C15—C16—C17^ii^	−178.5 (4)
C1—N1—C5—C4	1.2 (7)	C16—C15—C17—C16^ii^	0.8 (7)
Co1—N1—C5—C4	−170.8 (4)	C14—C15—C17—C16^ii^	178.5 (4)
C3—C4—C5—N1	0.8 (7)		

Symmetry codes: (i) −*x*, −*y*, −*z*; (ii) −*x*+1, −*y*, −*z*.

### Hydrogen-bond geometry (Å, °)

**Table:**

*D*—H···*A*	*D*—H	H···*A*	*D*···*A*	*D*—H···*A*
O3—H3A···N2^iii^	0.93	1.90	2.820 (5)	171
O3—H3B···O2^i^	1.03	1.71	2.704 (4)	161

Symmetry codes: (iii) −*x*+1, *y*−1/2, −*z*+1/2; (i) −*x*, −*y*, −*z*.
